# Intra- and Interrater Reliability of Infrared Image Analysis of Facial Acupoints in Individuals with Facial Paralysis

**DOI:** 10.1155/2020/9079037

**Published:** 2020-03-03

**Authors:** Xulong Liu, Jinghui Feng, Jingmin Luan, Chenxi Dong, Hefei Fu, Zhenying Wu

**Affiliations:** ^1^Department of Biomedical Engineering, School of Computer and Communication Engineering, Northeastern University, Qinhuangdao 066004, Hebei Province, China; ^2^Department of Acupuncture and Massage, Qinhuangdao Hospital of Traditional Chinese Medicine, Qinhuangdao 066004, Hebei Province, China

## Abstract

Infrared thermography (IRT), as a noncontact tool for temperature measurement, is widely applied in the study of acupuncture modernization. The aim of this study was to assess the intra- and interrater reliability of infrared image analysis of facial acupoints of subjects with facial paralysis and determine the factors influencing the variability of the measured values. A total of 26 patients with facial paralysis on one side, aged 26 to 53 years, participated voluntarily in the study. Facial infrared thermal images of all participants were analyzed by two trained raters at two different time points at a one-week interval. The intraclass correlation coefficient (ICC) was used to determine the intra- and interrater reliability of IRT measurements. The ICC values varied depending on the analyzed acupoints. The reliability of temperature measurement ranged from moderate to excellent (intrarater, ICC ranged from 0.669 to 0.990; interrater, ICC ranged from 0.661 to 0.987). The reliability of temperature difference measurement ranged from low to excellent (intrarater, ICC ranged from 0.412 to 0.882; interrater, ICC ranged from 0.334 to 0.828). The main influencing factor of reliability is the incomplete consistency in selecting acupoint positions when repeatedly positioning the same acupoint manually. Despite low reliability of temperature difference measurement at some acupoints, some auxiliary measures can be used to reduce the error of manual positioning. Thus, infrared thermal imaging still has the potential to assist in objective and quantitative research on acupuncture.

## 1. Introduction

Acupuncture, as an alternative medical treatment, is commonly used in the treatment of pain, arthritis, and facial paralysis, etc. in clinical practice of traditional Chinese medicine (TCM) by stimulating certain specific acupoints [1, 2]. However, the uniqueness of acupoint theory further hinders the spread and application of acupuncture in other countries. Therefore, it is necessary to apply modern medical imaging methods to study the specificity of acupoints. In recent years, infrared thermography (IRT), as a noncontact and nonradiative method for estimating skin temperature of the human body, has been widely used to assess peripheral effects of acupuncture and assist in the diagnosis of some diseases by measuring temperature changes at acupuncture points under certain physiological conditions [3, 4]. Litscher et al. [5] applied a thermal imaging camera to measure skin temperature (*T*_sk_) distribution at acupoints during acupuncture treatment in 10 healthy subjects. The authors observed that there was significant increase in temperature at a region of interest (ROI) around the Dazhui (GV14) acupoint. Lin et al. [6] used IRT to monitor skin temperature changes of human body in 36 healthy female subjects at acupoints during moxibustion and discovered that the maximum temperature increased by 11°C at the SP6 acupoint. With the application of IRT, Raith et al. [7] studied the rising phenomenon of skin temperature at Hegu (LI4) during laser acupuncture therapy. Huang et al. [8] used IRT to estimate skin temperature in the area of Zusanli (ST36) and observed that different acupuncture intensities had significant differences in skin temperature changes. By analyzing facial thermal images of patients with facial paralysis, Liu et al. [9–11] found that the bilateral temperature differences in acupoint areas on the left and right sides of face was greater in patients with facial paralysis than those of the healthy population, indicating that lesion severity was positively correlated with side-to-side temperature difference (Δ*T*). Zhang [12] presented a method of objective acupoint selection based on IRT in the treatment of facial paralysis. By comparing the skin temperature distribution on the left and right sides of face in patients, the acupoints with Δ*T* greater than 0.5°C were selected to apply acupuncture treatment, so as to achieve objectification and standardization of acupoints selected for acupuncture treatment of facial paralysis. Vardasca et al. used IRT to evaluate the facial thermal symmetry of subjects with orofacial pain before and after acupuncture treatment, and they found that using IRT to evaluate the effect of acupuncture treatment is objective and repeatable [13, 14].

These studies mentioned above exhibit the necessity to measure temperature in some specific acupoint areas, but there is still no specific software for automatic temperature measurement of human body, especially in the acupoint regions. Most of the above studies used standard software developed by the manufacturers of infrared thermal imagers that has high frequency of use in industrial or architectural fields [15]. Therefore, when IRT is utilized for temperature measurement in the acupoint area, the examiner is first obliged to locate the acupoint manually based on their experience, followed by temperature value reading. Deviation in manual location often leads to the inconsistency of repeated measurement results, and it is possible to obtain different temperature values at the same acupoint of the same thermal image by different examiners. Meanwhile, the same examiner may get different temperature values at different time points as well. Therefore, it is pretty necessary to evaluate the reliability of temperature measurement in acupoint areas using IRT.

Extensive studies have been reported concerning the reliability of local skin temperature measurement by IRT in healthy population and in patients with certain diseases. Zaproudina et al. [16] and Fernández-Cuevas et al. [17] applied IRT to measure skin temperature in some ROIs of healthy and overweight individuals, in combination with the analysis of its reliability and reproducibility. Corresponding results showed that IRT was a reliable tool for human body temperature measurement, regardless of healthy or overweight populations. Furthermore, IRT was also utilized by McCoy et al. [18] in their study to measure temperature of paravertebral muscles in healthy individuals, and its reliability was evaluated as excellent. Costa et al. [19] proposed two kinds of IRT analyses about masticatory and upper trapezius muscles. The intra- and interrater reliabilities were excellent in both healthy individuals and patients with temporomandibular disorder. Furthermore, Dibaifilho et al. [20] carried out a study focusing on the reliability of IRT of upper trapezius muscle in 24 subjects and applied three different methods to measure temperature in the myofascial trigger points. The intraclass correlation coefficient (ICC) was calculated to be ranged from 0.591 to 0.993. Rossignoli et al. [21] analyzed the reliability of local skin temperature measurement for wheelchair users via IRT. The results showed that temperature measurement with IRT could be used in related studies involving wheelchair users, but its reliability was variable depending mainly on the locations of the ROIs to be analyzed.

There have been studies emphasized on the reliability of temperature measurement by IRT in some ROIs of human body. As far as we know, there is, however, no reliability study of IRT temperature measurement in acupoint areas of human body, especially in patients with Bell's palsy. Bell's palsy is a type of facial paralysis with acute onset and unknown etiology, which often induces motor dysfunction of muscle group of facial expression, with the symptoms of facial paralysis varying from mild to severe [22]. In the field of alternative medicine, acupuncture is one of the most common treatments for Bell's palsy [23]. As mentioned above, in some studies, the acupoints needed for the treatment of patients with Bell's palsy were selected by analyzing the side-to-side temperature difference in facial acupoint areas [9, 10, 12]. Nevertheless, facial symptoms of subjects may induce troubles in the temperature measurement of facial acupoints. Therefore, compared with normal populations, the study on reliability of acupoints regional analysis with IRT may reveal more new problems by taking facial infrared thermal images of patients with Bell's palsy as a database.

Therefore, in order to assess whether IRT could become an objective and reliable auxiliary tool in acupuncture research, the main purpose of this study was to evaluate the intra- and interrater reliability of IRT measurements in different facial acupoint regions and determine the factors inducing the variability of the observed readings, via analyzing facial thermal images in Bell's palsy patients as a study case.

## 2. Materials and Methods

### 2.1. Subjects

A total of 26 volunteers with Bell's palsy were recruited in Qinhuangdao Hospital of Traditional Chinese Medicine, including 11 men and 15 women (mean ± s.d.; age, 47.50 ± 12.25 years; weight, 65.57 ± 8.19 kg; body mass index, 23.92 ± 2.45 kg/m^2^). According to the screening of specialists, the inclusion criteria of all Bell's palsy patients were (1) age ≥18 years; (2) in accordance with the clinical diagnostic criteria of Bell's palsy, with acute onset and unilateral facial paralysis; (3) treatment within 3 days after onset and without acupuncture treatment. Exclusion criteria were (1) patients suffering from similar diseases such as herpes zoster, Lyme disease, stroke, and brain tumors; (2) patients with skin diseases, eye diseases, otorhinolaryngological disease, and trigeminal neuralgia that might change the distribution of facial temperature; and (3) patients who have had facial cosmetic surgery.

According to the Helsinki Declaration, all participants were fully informed of the research plan and potential risks before the start of the study and provided with signed informed consent forms. The study plan was approved by the Ethics Committee of Qinhuangdao Hospital of Traditional Chinese Medicine.

### 2.2. Acquisition of IRT Images

Facial IRT images of all subjects were collected in the acupuncture clinic of Qinhuangdao Hospital of Traditional Chinese Medicine. Under a controlled environment, the ambient temperature was kept at 23–24°C, the humidity was about 50–60%, and a slightly dark fluorescent lamp was used. There was no obvious air convection and strong thermal radiation source in the room. The facial IRT images of subjects were collected using a medical infrared thermal camera (WP-95, Beijing Optoelectronic Technology Co., Ltd. Beijing, China). Corresponding parameters were described as follows: FPA sensor size of 256 × 256, infrared band of 7.5–13 *μ*m, NETD of <50 mK, and the emissivity preset at 0.98 [24].

All subjects were informed to stop smoking, drinking alcoholic or caffeine-containing products, taking medicine, cosmetics application, and violent exercise within 12 hours before the acquisition of facial IRT. The time of collection was limited between 9 and 10 a.m. to avoid changes of temperature distribution in human body caused by changes in the physiological rhythm of human body [25]. Before the acquisition of facial IRT, subjects were informed to sit quietly in the test environment for 20 minutes to adapt to the room temperature. After the stability of moods, subjects were asked to sit at the place 1.2 m away from the infrared camera, with a curtain placed in the back, to obtain a background with uniformed temperature distribution. By looking straight ahead, subjects were photographed to obtain the infrared thermogram of the three sides of head (the frontal part, left side, and right side) by the infrared thermal imager, respectively. Subjects in each group were photographed two times with an interval of 2 minutes, from which one group of figures with the best imaging quality was selected for analysis using the IRT analysis software (MC2.1) equipped by the thermal imager. The thermal images in the other group were used as backups.

### 2.3. Analysis of IRT

**Figure 1:**
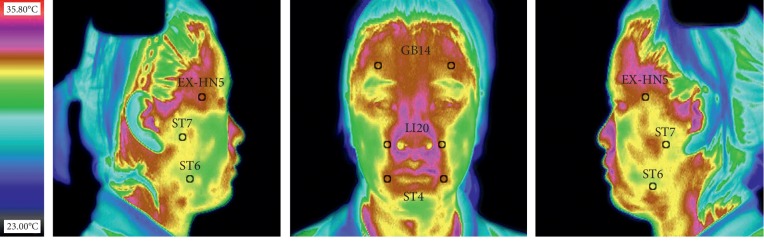
Distribution of the 12 regions of interest on the infrared thermography of the face.

A total of six facial acupoints (12 ROIs in total) were selected for the analysis in this study (Figure 1). These six acupoints were the most commonly used acupoints in the research of IRT-assisted acupuncture therapy for facial paralysis [12], which were Yangbai (GB14), Dicang (ST4), Yingxiang (LI20), Taiyang (EX-HN5), Jiache (ST6), and Xiaguan (ST7). The location of these acupoints on IRT followed the WHO standard, mainly depending on anatomical marks related to human body surface [26]. Each of these selected acupoints was distributed in pairs on the left and right sides in face, and these acupoints were thus used as the center to divide facial thermography of patients with Bell's palsy into 12 circular ROIs. There were 6 ROIs on frontal part of IRT, and 3 ROIs on the left and right sides of the IRT, respectively. The area of each ROI was about 29 pixels.

In this study, average temperature value (*T*_roi_) was used to represent the temperature of each ROI. *T*_roi_ refers to the average value of temperature of all pixels in the selected ROI. In order to analyze the intra- and interrater reliability, four analyses were carried out in 78 infrared thermograms (26 groups in total, 3 thermal images in each group) by two trained raters at the interval of 7 days between two different time points. At each time point, two raters measured the *T*_roi_ of each ROI in each infrared thermogram and the temperature difference (Δ*T*) of bilateral ROIs.

### 2.4. Statistical Analysis

Data normal distribution was verified by a Kolmogorov–Smirnov test, and all data met the assumption of normality. ANOVA for repeated measures was used to analyze the measurement outcomes between the two raters and between the two sessions. The intraclass correlation coefficient (ICC, two-way mixed model) was used to evaluate the intra- and interrater reliability and coefficient of variation (CV; SD/mean *∗* 100) to analyze the measurement variability. In addition, Bland–Altman plots were used to show the intra- and intraexaminer agreement and the dispersion of all observations. Statistical analysis was performed using SPSS software, version 20.0 (Chicago, IL, USA). Interpretation of ICC values was based on that suggested by Fleiss [20]. For values less than 0.40, the reliability was considered low; between 0.40 and 0.75, moderate; between 0.75 and 0.90, substantial; and finally, values greater than 0.90, excellent.

## 3. Results

Of the 26 subjects, 17 had right-sided facial paralysis and 9 had left-sided facial paralysis. To analyze the intra- and interrater reliability, two raters assessed the facial IRT of the same group of Bell's palsy patients at two different time points. Tables 1 and 2 describe the results of repeated measurements for 26 subjects. As illustrated, the mean temperature range of facial acupoints was 32.31∼35.04°C in Bell's palsy patients. Besides, the temperature value extracted from IRT of the left and right sides (Taiyang, Jiache, and Xiaguan) was generally lower than that of the frontal part (Yangbai, Yingxiang, and Dicang). Meanwhile, the Δ*T* values of the left and right sides were commonly higher than those of the frontal part (see Table 2).

ICC and CV values of intra- and interrater in the acupoint temperature measurement are shown in Tables 3 and 4. ICC values of intrarater ranged from 0.669 to 0.990, and the minimum value of 0.669 appeared at Xiaguan, with a moderate reliability level. Besides, CV values distributed between 1.98 and 3.27, and the maximum value of 3.27 appeared at the acupoint of Jiache. Furthermore, ICC values of interrater ranged from 0.661 to 0.987, and the lowest value of 0.661 occurred at Xiaguan, with a moderate reliability level. CV values ranged from 2 to 3.30, and the maximum value of 3.30 appeared at the acupoint of Jiache.

Table 5 illustrates the ICC values of intra- and interrater reliability of facial acupoint Δ*T* measurement. The ICC value of intrarater reliability ranged from 0.412 to 0.882, and the minimum value of 0.412 appeared at Xiaguan, with a moderate reliability level. The range of ICC of interrater reliability was 0.334 to 0.828, of which, the ICC values of the three acupoints of Taiyang, Jiache, and Xiaguan showed low reliability of 0.336, 0.356, and 0.334, respectively.

**Figure 2:**
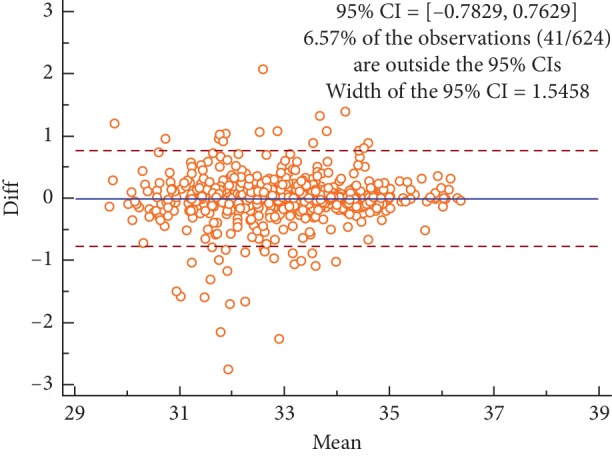
Bland–Altman plot for intrarater agreement between two sets of the temperature values collected during the two sessions, 6.57% of all readings done fell outside the 95% agreement limits.

**Figure 3:**
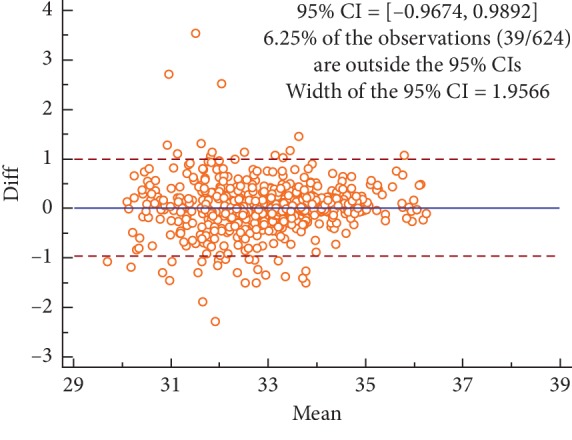
Bland–Altman plot for interrater agreement between two sets of the temperature values collected by two different raters, 6.25% of all readings done fell outside the 95% agreement limits.

**Figure 4:**
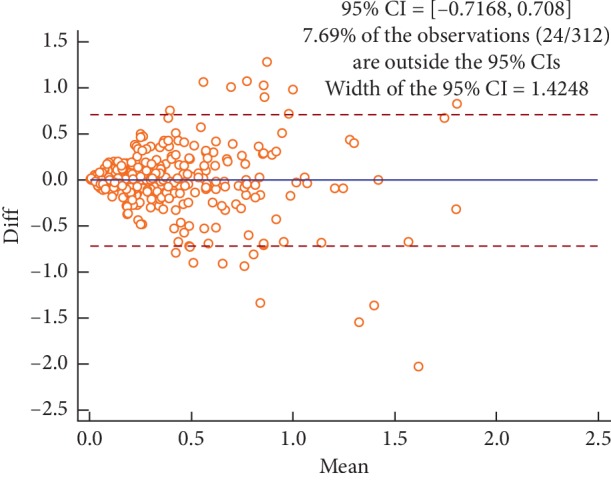
Bland–Altman plot for intrarater agreement between two sets of the side-to-side temperature differences (Δ*T*) collected during the two sessions, 7.69% of all readings done fell outside the 95% agreement limits.

**Figure 5:**
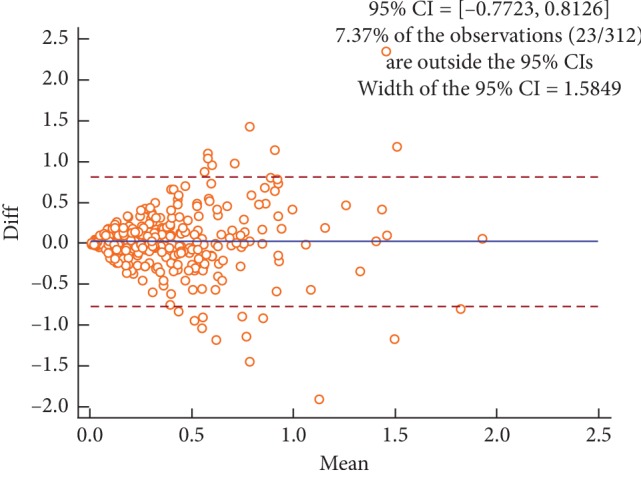
Bland–Altman plot for interrater agreement between two sets of the side-to-side temperature differences (∆*T*) collected by two different raters, 7.37% of all readings done fell outside the 95% agreement limits.

Figures 2–5 are the Bland–Altman difference plots, which were used to visualize the consistency between the two sets of measurements. Among these figures, two sets of data compared in Figures 2 and 3 were derived from the temperature values of each acupoint area in 26 subjects. Besides, the data used in Figures 4 and 5 were the Δ*T* values in 26 subjects. Figures 2 and 4 depict the consistency of intrarater between the two different time points, among which 6.57% and 7.69% of all data distributed outside the 95% agreement limits, respectively. Figures 3 and 5 show the consistency between two interraters at the same time, 6.25% and 7.37% of all data spread outside the 95% agreement limits, respectively.

Temperature difference between the two bilateral ROIs greater than 0.5°C is generally considered to be a sign of local physiological abnormalities in human body [12, 15]. Therefore, 0.5°C is used as a threshold value to evaluate the inconsistency among results of repeated temperature measurements. The inconsistency among repeated measurements of temperature at acupoint is that the absolute value of the difference is greater than 0.5°C between the two measurements. The inconsistency among repeated measurement of side-to-side temperature difference is that the measured value is less than 0.5°C in the first time and greater than or equal to 0.5°C in the second time. As shown in Table 6, whether it was temperature measurement or temperature difference measurement, the inconsistency rate of repeated temperature measurement of acupoints (Yangbai, Yingxiang, Dicang) with IRT on the frontal part was less than that (Taiyang, Jiache, Xiaguan) on the left and right sides.

## 4. Discussion

As a noncontact temperature measuring tool, IRT has been applied widely in the research on the modernization of acupuncture. In these studies, IRT is generally applied for skin temperature measurement in acupoint areas. In the process of temperature measurement, it is a common phenomenon to analyze the same IRT by different raters or a rater at different time points. However, it may lead to the inconsistency between data obtained in repeated measurements, which will in turn decrease the reliability of corresponding results. In addition, the use of IRT to measure temperature of facial acupoints in patients with Bell's palsy can assist the acupuncture treatment of Bell's palsy. Nevertheless, the research on reliability has not yet been reported concerning the IRT analysis in patients with Bell's palsy so far. Therefore, using infrared thermogram analysis in patients with Bell's palsy, the main purpose of this research was to evaluate the intra- and interrater reliability in the measurement of skin temperature in acupoint areas and side-to-side temperature difference measurement, so as to determine the main factors that might induce data inconsistencies in repeated measurements, thereby explaining the specificity of temperature values of different acupoints more credibly.

In this research, six acupoints commonly used in the treatment of Bell's palsy were selected as the ROIs for analysis, including Yangbai, Yingxiang, Dicang, Taiyang, Jiache, and Xiaguan. At these acupoints, Yangbai, Yingxiang, and Dicang are located in the infrared thermogram on frontal part of the face, and Taiyang, Jiache, and Xiaguan are located on the left and right sides of the face. At present, there is no software for automatic temperature measurement in acupoint areas of IRT; it is therefore necessary to extract the temperature values in different acupoints manually. However, the manual positioning accuracy is quite different due to the difference in the location of acupoints. Accordingly, in this research, it was suggested that the reliability level of facial acupoints temperature measurement varied with its location, and some measurement parameters (temperature, temperature difference, ICC, CV) on acupoints of the frontal part were different from those of the lateral face.

Temperature distribution in the facial region of all subjects showed a model of high temperature in the middle region and low temperature on both sides. The average temperature of each acupoint on the IRT on frontal part of the face (Yangbai, Yingxiang, and Dicang) was higher than that on the right and left sides of the face (Taiyang, Jiache, and Xiaguan), as shown in Table 1. This result was similar to the conclusion drawn by Guan et al.; to be specific, both patients with Bell's palsy and healthy people presented a T-type hot zone on the facial IRT [27]. The temperature of the forehead, the canthus, the nose wings, and the mouth corners is relatively high, and the temperature of the cheeks on both sides is relatively low, which is mainly related to the vascular distribution in the face. The Δ*T* between facial acupoints on the left and right sides of patients with Bell's palsy was generally greater than that in the frontal acupoints (as shown in Table 2). Furthermore, the average value of side-to-side temperature difference was 0.51°C at the three acupoints on IRT of lateral face, while that of frontal part was 0.28°C. Also, some of the previous studies conducted by Wu et al. [10] have drawn similar conclusions, but in their researches, the side-to-side temperature difference between the two acupoints of Yangbai (GB14) and Yingxiang (LI20) was greater than that of the results in the present study. Potential reason might be associated with the different numbers of subjects, as well as the difference of facial paralysis severity of selected subjects between those researches and the present research.

The degree of intra- and interrater reliability varied with the location of acupoints during temperature measurement in different acupoint areas, as shown in Tables 3 and 4. The ICC values ranged from 0.661 to 0.990, and corresponding reliability degree exhibited obvious improvement from moderate to excellent. Among them, the reliability degree of the three points on frontal part was excellent, while that on the lateral face varied from moderate to excellent. Besides, in view of CV values in Tables 3 and 4, the discreteness of temperature values on acupoints of lateral face (mean, CV = 3.1) was higher than that on frontal part (mean, CV = 2.1). The main reason for the above phenomenon was that the accuracy of manual localization was different for different acupoints, in which the accuracy of the three acupoints on the frontal part was higher than that on the lateral face.

The ICC values ranged from 0.334 to 0.882 via the measurement of side-to-side temperature differences at different acupoints, with the reliability degree ranging from low to excellent, which was similar to that of the preceding conclusion. The ICC values of side-to-side temperature differences measured at acupoints on the lateral face were lower than that on the frontal part. In addition, low reliability was found in the side-to-side temperature difference measurement of acupoints on the lateral face. Therefore, when using IRT to measure the side-to-side temperature differences at acupoints on the lateral face, auxiliary measures should be adopted to improve the reliability, for example, sticking labels beside ROI, etc. Costa et al. [19] stuck labels on the facial temporalis and near the masseter to facilitate more accurate location of the ROIs to be analyzed. The ICC values of the intra- and interraters were all greater than 0.990 in their research results. In this study, the two acupoints of Taiyang and Jiache were close to the location of ROIs mentioned above, but there were no adhesive labels, and ICC values were less than 0.8, which were obviously lower than that reported in the study performed by Costa et al. [19]. Although sticking labels at the side of ROI can reduce manual error and improve reliability, this method was not used in this research. The main reason lied in that there were excessive labels and patients with Bell's palsy themselves might have antagonism on their facial labels, which could both affect the temperature distribution of human skin.

The average ICC value of side-to-side temperature difference measurement on facial acupoints was lower than that of acupoint temperature measurement. Besides, a similar conclusion also can be drawn from the Bland–Altman plots (Figures 2–5) that the intra- and interrater consistency of acupoint temperature measured were greater than that of side-to-side temperature difference. The above conclusions were similar to those of McCoy et al. [18] and different from those of Fernández-Cuevas et al. [17]. The cause of this phenomenon might be that temperature difference measurement required measuring the temperature values of the two bilateral ROIs. This produced more random errors than that of single ROI temperature measurement. However, Fernández-Cuevas and others used the software to locate ROIs automatically, which greatly reduced the random error caused by the inconsistency of manually location. Therefore, the ICC values of temperature difference measurement reported by Fernández-Cuevas et al. were in fair agreement with that of temperature measurement.

For normal human body, the skin temperature distribution of body surface is symmetric bilaterally, that is, the side-to-side temperature difference is less than 0.3°C, 0.4°C, or 0.5°C [13, 16]. Therefore, Δ*T* over 0.5°C is generally regarded as a sign of physiological abnormalities [12]. In the acupuncture treatment of Bell's palsy, it can be used as a standard for the selection of acupoints needed for treatment. Therefore, 0.5°C was selected as a threshold to assess the inconsistency between repeated measurements in this research. In Table 6, the inconsistency rates of the two sets of data obtained from repeated measurements of three acupoints on the lateral face were both higher than that of three acupoints on the frontal part, whether in temperature measurement or temperature difference measurement. The reason was the same as above, which was mainly caused by varied degree of difficulty of manual localization in different acupoint areas. In addition, in the Δ*T* measurement at different acupoints, 10.2% of the Δ*T* values were not consistent in the two intrarater measurements in average, and there was an average of 15% inconsistency in the Δ*T* values in the two interrater measurements. In other words, in two measurements of the same infrared thermogram, owing to human factors, Δ*T* value changed from less than 0.5°C to 0.5°C or more or showed the reverse change trend. It also indicated that the same patient may receive inconsistent acupoint selection scheme due to human factors in the treatment of Bell's palsy. Therefore, it was held that even excellent reliability results have been obtained in the research of IRT, different thresholds should also be set depending on different physiological conditions of subjects to carry out the consistency analysis of repeated measurement data in this research.

Several factors may exert influence on the temperature distribution of body surface, such as external environment, subcutaneous fat content, sweating, and illness [15], which thus should be controlled in the process of IRT analysis. In this research, the standard process was used to collect infrared thermogram of subjects under controlled environment, so as to avoid interference from changes in the external environment on IRT analysis. Furthermore, in the selection of subjects, relevant criteria were predetermined as patients with Bell's palsy, without the inclusion of patients with BMI over 25 to avoid the effects of human physiological parameters on the reliability analysis.

This research focused on the intra- and interrater reliability analysis in temperature measurement of infrared thermogram in human acupoint areas and mainly assessed the impacts of human factors on the reliability by raters. Accordingly, considering that the reproducibility of temperature measurement in acupoint areas is mainly affected by the change of physiological state of human body, and the reproducibility was not analyzed in this research. In the future research, the reproducibility of temperature measurement through IRT in patients with facial paralysis will be further analyzed by collecting two groups of infrared thermograms from the same subjects at intervals of five seconds or one day under the condition of limiting facial paralysis severity of subjects. In addition, considering that there have been extensive researches on the reliability of infrared thermograms of healthy population, the reliability of acupoint temperature measurement in healthy subjects was not analyzed in this research. In future studies, comparison will be carried out between healthy subjects and patients with facial paralysis, the ROI will be further expanded to acupoint areas of other parts of the body, and the reliability will also be evaluated regarding the *T*_max_ [28] temperature representation method.

## 5. Conclusion

Results in this research suggest that the intra- and interrater reliability level of temperature measurement of IRT is acceptable in facial acupoints on patients with facial paralysis. However, there is low reliability in results of side-to-side temperature difference measurement on both sides of the face. In addition, whether in temperature measurement or in temperature difference measurement, the inconsistency rates of the two measurement values of acupoints on the lateral face are both higher than that on the frontal acupoints, which is correlated with the reason that the accuracy of the manual location of acupoints on the lateral face is lower than that of the frontal acupoints.

There are different reliability values in analyses of IRT on different acupoint areas, which is depended on acupoints location. Although the reliability level of the measurement of side-to-side temperature difference is relatively unsatisfied in some acupoints, the IRT still has the potential to be an objective and quantitative assessment tool for acupuncture treatment scheme of facial paralysis through the implementation of some auxiliary measures to reduce artificial error.

## Figures and Tables

**Table 1:** Mean and standard deviation results of acupoint temperature values from 26 participants in two sessions collected by two different raters.

Acupoint	Rater 1, *T*_sk_ (mean ± SD)		Rater 2, *T*_sk_ (mean ± SD)	
	Session 1	Session 2	Session 1	Session 2
L GB14	34.52 ± 0.77	34.51 ± 0.76	34.52 ± 0.74	34.55 ± 0.76
R GB14	34.54 ± 0.84	34.56 ± 0.86	34.56 ± 0.69	34.68 ± 0.76
L LI20	34.65 ± 0.74	34.66 ± 0.76	34.87 ± 0.73	34.98 ± 0.73
R LI20	34.82 ± 0.71	34.76 ± 0.72	34.91 ± 0.69	35.04 ± 0.70
L ST4	34.32 ± 0.79	34.31 ± 0.79	34.30 ± 0.79	34.34 ± 0.79
R ST4	34.54 ± 0.71	34.46 ± 0.67	34.49 ± 0.75	34.46 ± 0.72
L EX-HN5	33.12 ± 0.94	33.34 ± 0.82	33.02 ± 0.85	33.08 ± 0.79
R EX-HN5	33.29 ± 0.83	33.35 ± 0.78	33.29 ± 0.70	33.39 ± 0.74
L ST6	32.37 ± 1.06	32.31 ± 1.08	32.78 ± 1.07	32.56 ± 0.94
R ST6	32.54 ± 0.96	32.63 ± 0.95	32.82 ± 0.96	32.75 ± 0.94
L ST7	33.16 ± 0.78	33.14 ± 0.78	33.07 ± 0.83	32.64 ± 1.02
R ST7	33.55 ± 0.72	33.56 ± 0.76	33.19 ± 0.69	32.97 ± 0.72

**Table 2:** Comparison of mean and standard deviation of side-to-side temperature difference absolute values of facial acupoints from 26 participants with Bell's palsy.

Acupoint	Rater 1, Δ*T* (Mean ± SD)		Rater 2, Δ*T* (Mean ± SD)	
	Session 1	Session 2	Session 1	Session 2
GB14	0.27 ± 0.20	0.35 ± 0.30	0.25 ± 0.20	0.27 ± 0.23
LI20	0.27 ± 0.26	0.24 ± 0.20	0.23 ± 0.20	0.22 ± 0.21
ST4	0.34 ± 0.28	0.36 ± 0.29	0.33 ± 0.32	0.33 ± 0.29
EX-HN5	0.33 ± 0.41	0.37 ± 0.39	0.55 ± 0.44	0.47 ± 0.37
ST6	0.42 ± 0.33	0.51 ± 0.48	0.68 ± 0.65	0.48 ± 0.34
ST7	0.61 ± 0.46	0.62 ± 0.48	0.54 ± 0.45	0.55 ± 0.43

**Table 3:** Intrarater reliability of temperature measurement of facial acupoints from 26 participants with Bell's palsy.

Acupoint	Rater 1			Rater 2		
	CV (%)	ICC	Scale	CV (%)	ICC	Scale
L GB14	2.19	0.989	Excellent	2.15	0.990	Excellent
R GB14	2.42	0.986	Excellent	2.07	0.952	Excellent
L LI20	2.13	0.916	Excellent	2.08	0.960	Excellent
R LI20	2.04	0.959	Excellent	1.99	0.946	Excellent
L ST4	2.29	0.964	Excellent	2.28	0.972	Excellent
R ST4	1.98	0.941	Excellent	2.12	0.860	Substantial
L EX-HN5	2.64	0.846	Substantial	2.46	0.745	Moderate
R EX-HN5	2.39	0.906	Excellent	2.14	0.873	Substantial
L ST6	3.27	0.986	Excellent	3.08	0.874	Substantial
R ST6	2.90	0.969	Excellent	2.86	0.701	Moderate
L ST7	2.34	0.915	Excellent	2.88	0.669	Moderate
R ST7	2.19	0.861	Substantial	2.13	0.714	Moderate

CV : coefficient of variation; ICC : intraclass correlation coefficient.

**Table 4:** Interrater reliability of temperature measurement of facial acupoints from 26 participants with Bell's palsy.

Acupoint	Session 1			Session 2		
	CV (%)	ICC	Scale	CV (%)	ICC	Scale
L GB14	2.16	0.981	Excellent	2.18	0.987	Excellent
R GB14	2.19	0.936	Excellent	2.32	0.935	Excellent
L LI20	2.11	0.912	Excellent	2.16	0.922	Excellent
R LI20	2.00	0.942	Excellent	2.06	0.946	Excellent
L ST4	2.29	0.963	Excellent	2.28	0.942	Excellent
R ST4	2.09	0.875	Substantial	2.00	0.951	Excellent
L EX-HN5	2.68	0.842	Substantial	2.43	0.842	Substantial
R EX-HN5	2.27	0.823	Substantial	2.25	0.818	Substantial
L ST6	3.30	0.704	Moderate	3.11	0.765	Substantial
R ST6	2.94	0.773	Substantial	2.86	0.850	Substantial
L ST7	2.42	0.719	Moderate	2.84	0.848	Substantial
R ST7	2.16	0.661	Moderate	2.39	0.718	Moderate

CV : coefficient of variation; ICC : intraclass correlation coefficient.

**Table 5:** Intra- and interrater reliability of temperature difference measurement of bilateral facial acupoints from 26 participants with Bell's palsy.

Acupoint	Intrarater reliability				Interrater reliability			
	Rater 1		Rater 2		Session 1		Session 2	
	ICC	Scale	ICC	Scale	ICC	Scale	ICC	Scale
GB14	0.882	Substantial	0.830	Substantial	0.721	Moderate	0.710	Moderate
LI20	0.466	Moderate	0.595	Moderate	0.828	Substantial	0.523	Moderate
ST4	0.768	Substantial	0.666	Moderate	0.650	Moderate	0.810	Substantial
EX-HN5	0.558	Moderate	0.643	Moderate	0.336	Low	0.423	Moderate
ST6	0.798	Substantial	0.421	Moderate	0.477	Moderate	0.356	Low
ST7	0.706	Moderate	0.412	Moderate	0.334	Low	0.599	Moderate

ICC : intraclass correlation coefficient; CI : confidence interval.

**Table 6:** The inconsistent rate of temperature values of facial acupoints and temperature difference values between left and right acupoints in patients with Bell's palsy during repeated measurements.

Acupoint	Intrarater disagreement		Interrater disagreement	
	*T*_sk1-2_ > 0.5	Δ*T*_1-2_ > 0.5	*T*_sk1-2_ > 0.5	Δ*T*_1-2_ > 0.5
GB14	1.92% (2/104)	1.92% (7/52)	3.85% (4/104)	19.2% (10/52)
LI20	5.77% (6/104)	5.77% (3/52)	15.4% (16/104)	17.3% (9/52)
ST4	9.62% (10/104)	19.2% (10/52)	3.85% (4/104)	17.3% (9/52)
EX-HN5	19.2% (20/104)	25% (13/52)	20.2% (21/104)	34.6% (18/52)
ST6	19.2% (20/104)	32.7% (17/52)	32.7% (34/104)	30.8% (16/52)
ST7	22.1% (23/104)	23.1% (12/52)	48.1% (50/104)	25% (13/52)

Note: Subscript 1-2 represents the first measurement and the second measurement. The Tsk of each acupoint area contains skin temperature values in two regions of interest (ROIs) on the left and right sides of the face. Δ*T* represents the absolute value of the temperature difference between the two ROIs on the left and right sides of an acupoint. In this study, the inconsistency between the two *T*_sk_ values in two repeated measurements is defined as that the absolute value of the difference between the two measurements is greater than 0.5°C. The inconsistency between the two Δ*T* values is defined as a Δ*T* of less than 0.5°C, while the other measured Δ*T* is greater than 0.5°C. *T*_sk1-2_ > 0.5 and Δ*T*_1-2_ > 0.5 refers to the ratio between the number of inconsistent measurements and the total number of measurements in the repeated measurements.

## Data Availability

The relevant raw data can be obtained by contacting the first author or corresponding author.

## References

[1] Vickers A. J., Vertosick E. A., Lewith G. (2018). Acupuncture for chronic pain: update of an individual patient data meta-analysis. *The Journal of Pain*.

[2] Yanq X., Ye Y., Xia Y. (2015). A precise and accurate acupoint location obtained on the face using consistency matrix pointwise fusion method. *Journal of Traditional Chinese Medicine*.

[3] Litscher G. (2009). Ten years evidence-based high-tech acupuncture-a short review of peripherally measured effects. *Evidence-Based Complementary and Alternative Medicine*.

[4] She Y., Ma L., Zhu J. (2017). Comparative study on skin temperature response to menstruation at acupuncture points in healthy volunteers and primary dysmenorrhea patients. *Journal of Traditional Chinese Medicine*.

[5] Litscher G., Wang L., Huang T., Zhang W. (2011). Violet laser acupuncture-part 3: pilot study of potential effects on temperature distribution. *Journal of Acupuncture and Meridian Studies*.

[6] Lin L. M., Wang S. F., Lee R. P., Hsu B. G., Tsai N. M., Peng T. C. (2013). Changes in skin surface temperature at an acupuncture point with moxibustion. *Acupuncture in Medicine: Journal of the British Medical Acupuncture Society*.

[7] Raith W., Litscher G., Sapetschnig I. (2012). Thermographical measuring of the skin temperature using laser needle acupuncture in preterm neonates. *Evidence-Based Complementary and Alternative Medicine*.

[8] Huang T., Huang X., Zhang W., Jia S. Y., Cheng X. N., Litscher G. (2013). The influence of different acupuncture manipulations on the skin temperature of an acupoint. *Evidence-Based Complementray and Alternative Medicine*.

[9] Liu X. L., Hong W. X., Zhang T., Wu Z. Y., Zhang D. (2011). Anomaly of infrared thermal radiation intensity on unilateral mild to moderate bell’s palsy. *Spectroscopy and Spectral Analysis*.

[10] Wu Z. Y., Liu X. L., Hong W. X., Zhang D. (2010). Research on the correlation between the temperature asymmetry at acupoints of healthy and affected side and the severity index of facial paralysis. *Chinese Acupuncture & Moxibustion*.

[11] Liu X. L., Fu B. R., Xu L. W., Lu N., Yu C. Y., Bai L. Y. (2016). Automatic assessment of facial nerve function based on infrared thermal imaging. *Spectroscopy and Spectral Analysis*.

[12] Zhang D. (2007). A method of selecting acupoints for acupuncture treatment of peripheral facial paralysis by thermography. *The American Journal of Chinese Medicine*.

[13] Vardasca R., Ring E. F. J., Plassmann P., Jones C. D. (2012). Thermal symmetry of the upper and lower extremities in healthy subjects. *Thermology International*.

[14] Vardasca R., Pais-Clemente M., Pinto A., Gabriel J. The outcomes of thermal symmetry after orofacial pain acupuncture treatment.

[15] Fernández-Cuevas I., Bouzas Marins J. C., Arnáiz Lastras J. (2015). Classification of factors influencing the use of infrared thermography in humans: a review. *Infrared Physics & Technology*.

[16] Zaproudina N., Varmavuo V., Airaksinen O., Närhi M. (2008). Reproducibility of infrared thermography measurements in healthy individuals. *Physiological Measurement*.

[17] Fernández-Cuevas I., Marins J. C., Carmona P. G. (2012). Reliability and reproducibility of skin temperature of overweight subjects by an infrared thermography software designed for human beings. *Thermology International*.

[18] McCoy M., Campbell I., Stone P. (2011). Intra-examiner and inter-examiner reproducibility of paraspinal thermography. *PLoS One*.

[19] Costa A. C. S., Dibai Filho A. V., Packer A. C., Rodrigues-Bigaton D. (2013). Intra and inter-rater reliability of infrared image analysis of masticatory and upper trapezius muscles in women with and without temporomandibular disorder. *Brazilian Journal of Physical Therapy*.

[20] Dibaifilho A. V., Guirro E. C., Ferreira V. T. (2014). Reliability of different methodologies of infrared image analysis of myofascial trigger points in the upper trapezius muscle. *Brazilian Journal of Physical Therapy*.

[21] Rossignoli I., Benito P. J., Herrero A. J. (2014). Reliability of infrared thermography in skin temperature evaluation of wheelchair users. *Spinal Cord*.

[22] Gilden D. H. (2004). Bell’s palsy. *New England Journal of Medicine*.

[23] Li P., Qiu T., Qin C. (2015). Efficacy of acupuncture for Bell’s palsy: a systematic review and meta-analysis of randomized controlled trials. *PLoS One*.

[24] Steketee J. (1973). Spectral emissivity of skin and pericardium. *Physics in Medicine and Biology*.

[25] Reilly T., Brooks G. (1986). Exercise and the circadian variation in body temperature measures. *International Journal of Sports Medicine*.

[26] WHO Western Pacific Regional Publications (2009). *WHO Standard Acupuncture Point Locations in the Western Pacific Region*.

[27] Guan L., Li G., Yang Y., Deng X., Cai P. (2012). Infrared thermography and meridian-effect evidence and explanation in Bell’s palsy patients treated by moxibustion at the Hegu (LI4) acupoint Overall regulation or a specific target?. *Neural Regeneration Research*.

[28] Ludwig N., Formenti D., Gargano M., Alberti G. (2014). Skin temperature evaluation by infrared thermography: comparison of image analysis methods. *Infrared Physics & Technology*.

